# The Rad52 SSAP superfamily and new insight into homologous recombination

**DOI:** 10.1038/s42003-023-04476-z

**Published:** 2023-01-23

**Authors:** Ali Al-Fatlawi, Michael Schroeder, A. Francis Stewart

**Affiliations:** 1 Biotechnology Center, Center for Molecular and Cellular Bioengineering, Technische Universität Dresden, Tatzberg 47, 01307 Dresden, Germany; 2 School of Biotechnology and Biomolecular Sciences, University of New South Wales, Sydney, Australia

**Keywords:** DNA recombination, Structural biology

## Abstract

Recent structures of DNA-bound bacterial and phage recombinases provide insights into homologous recombination and suggest relation to the eukaryotic Rad52 and identification of a Rad52 single strand annealing protein (SSAP) superfamily.

## Teaser

Rad52 is a highly conserved eukaryotic protein that can mediate the annealing of complementary DNA strands to initiate homologous recombination for the repair of double-strand breaks^1^. Suspicions that at least some prokaryotic single-strand annealing proteins (SSAPs) are related to Rad52 have been discussed for more than two decades. Two recent cryo-EM structures^2,3^ now put the issue beyond doubt.

Homologous recombination (HR) is essential for all cellular life because it serves to rescue replication fork catastrophes and repair double-strand breaks^4^. HR is also utilized by many bacteriophages and molecular biologists for competitive fitness, in the latter case for recombineering and precise genome engineering. A vital early step in HR involves the pairing of complementary DNA strands, which is promoted by recombinases that bind single-stranded DNA in order to enhance the discovery of complementary regions and subsequently stabilize the discovery. Due to the importance of HR, the ubiquitous cellular HR recombinase, bacterial RecA and its eukaryotic homologue, Rad51, have been extensively studied^5^. The apparent diversity amongst other, non-RecA/Rad51, HR recombinases, broadly termed single-strand annealing proteins (SSAPs), was partly rationalized when three groups were identified within the diversity. The three groups were classified according to their most prominent members; Rad52, RecT/Redβ and Erf^6^. Electron microscopy imaging of the λ phage SSAP, Redβ, revealed spectacular un-/dodecameric rings^7^, which resonated with the rings displayed by human RAD52^8–10^ to suggest the intriguing possibility that SSAP action may have an underlying unity. Subsequently, advanced bioinformatic tools identified a very distant relationship amongst SSAPs based on three short motifs that are also the most conserved sequences in the Rad52 group, which led to the proposition that the SSAPs comprise a superfamily and are not merely functionally related^11–13^.

**Fig. 1:**
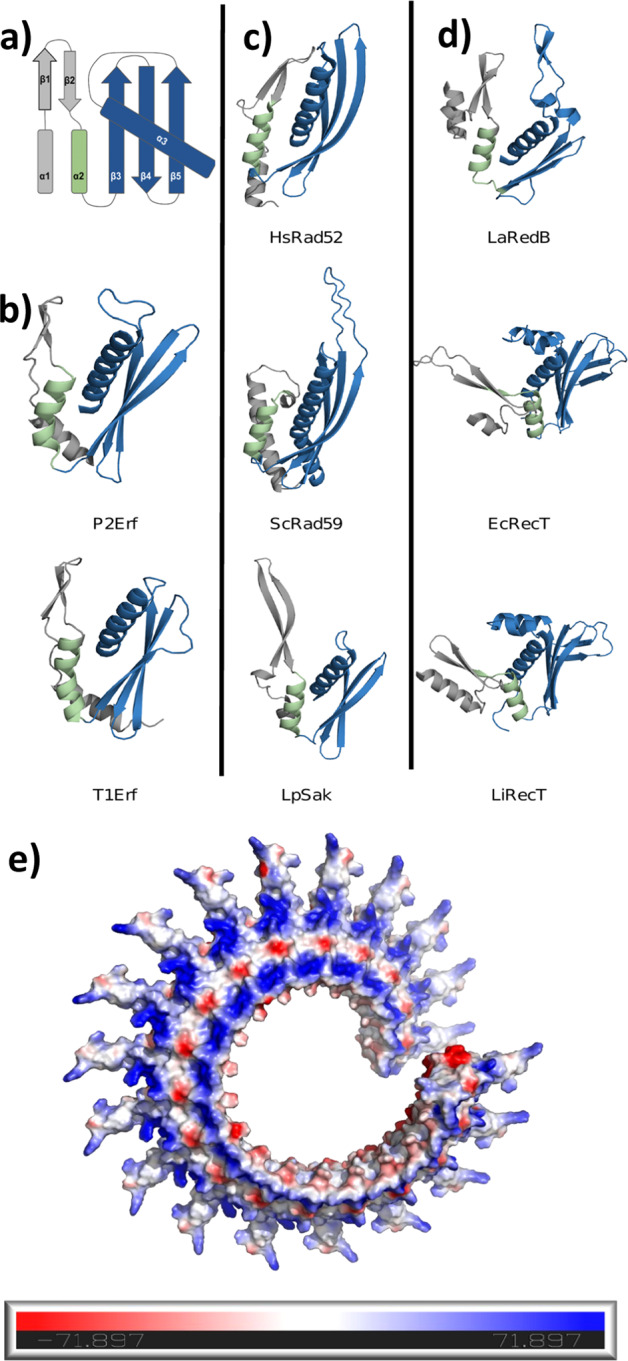
The Rad52 SSAP superfamily protein fold and AlphaFold predictions. **a** Diagram of the Rad52 SSAP protein fold secondary structure, using RAD52 secondary structural designations^9,10^, with the most conserved elements in blue and the most variable in grey. **b**–**d** Selected examples of the Rad52 SSAP fold from the three SSAP classes. **b** Erf: *E.coli* phage P2Erf, *E.coli* phage T1Erf. **c** Rad52: *H.sapiens* RAD52*, S.cerevisiae* Rad59; *L.lactis* phage ul36 SakRad52. **d** RecT/Redβ: λ phage Redβ, *E.coli* rac phage RecT; Li, *Listeria innocua* RecT. **e** AlphaFold projected full-length Redβ filament displaying electrostatic surfaces presenting the positively charged ssDNA binding groove (red) between negatively charged ridges (blue). The C-terminal three α-helical bundle^27^, which is not part of the annealing domain or the published cryo-EM structure^2^ but is required for HR^23^, is the perpendicular projection away from the helical axis of the filament.

However, securing the Rad52 SSAP superfamily hypothesis required more substantial evidence than a very faint sequence relationship or a propensity to multimerize, and several labs have pursued structural and mechanistic studies to resolve the issue. Although the structure of the human RAD52 annealing domain has been known for 20 years^9,10^, a representative structure from the diversity of bacterial and phage SSAPs has been lacking. Somewhat like London buses, two such structures have just arrived after decades of various labs being frustrated by inherent SSAP multimerization at the high in vitro concentrations required for X-ray crystallography and NMR. However, cryoEM is not disadvantaged by this problem and now exquisite annealed DNA filament structures for the annealing domain of λ phage Redβ^2^ and a full-length RecT from a *Listeria* phage^3^ have been solved. Despite the near-complete absence of amino acid sequence alignment, these structures are clearly similar to each other as well as to the known RAD52 structure. Thereby the RAD52 SSAP superfamily proposition is now conclusively established and a new class of protein fold has been identified (Fig. 1a).

The RAD52 SSAP superfamily protein fold involves five conserved elements: (i) an antiparallel three stranded β-sheet, which forms the inner surface of the helical filament; (ii) an α-helix (α3 in Fig. 1) that is packed across the three β-sheet strands. Together α3 and the β-sheet are the most conserved part of this protein fold (blue in Fig. 1) and set the inherent curvature of the filaments formed by multimerization; (iii) a second α-helix (α2, green) packs with α3; followed by (iv) a β-hairpin and (v) α-helix 1. Utilizing AlphaFold^14,15^ for SSAP modelling revealed that not only are other RecT/Redβ SSAPs based on the same design but also members of the Erf group (Fig. 1b), as previously anticipated^12^. All SSAPs contain the first three elements but variability in the other two elements (Fig. 1b, grey) is evident. Notably the most diverse Rad52 members, *S.c*.Rad59^16^ and the prokaryotic SakRad52^17^, appear to either lack the β-hairpin or present a greatly extended version of it. To further validate the Rad52 superfamily, we screened the AlphaFold library of one million structures using Foldseek^18^ with the five-element structure from HsRAD52. Of the 25 top hits, 18 were Rad52 variations (and another 5 were unknown proteins). Therefore, we conclude that the Rad52 SSAP fold is not common or previously identified and probably unique to SSAPs. This list of 25 plus a variety of AlphaFold SSAP structures for viewing by PyMOL can be found at this link https://sharing.biotec.tu-dresden.de/index.php/s/8cZI7i8EdZhENoN

The new bacteriophage filament structures not only secure the Rad52 SSAP superfamily hypothesis, but also deliver pioneering insight into SSAP annealing mechanisms, because no high-resolution structure of RAD52, or any other SSAP, bound to annealed DNA strands has been previously achieved. In concordance with a previous deduction^11^, the annealed DNA strands lie on the outside of the helical filament and the two strands do not cross each other with respect to the underlying protein helix. Therefore, despite Watson–Crick base pairing, the annealed strands must be more underwound than B-form dsDNA. This explains how SSAPs can be tightly bound to the annealed intermediate but have no, or little, affinity for B-form dsDNA.

Both bacteriophage structures show one DNA strand bound in a deep groove through electrostatic and hydrogen bonds to the phosphodiester backbone so the bases are presented outward. This groove is the same as the known RAD52 ssDNA binding groove^19^ and includes the only identifiable amino acid sequence signature in the Rad52 SSAP superfamily^11^. However, concordant with the substantial divergence of amino acid sequence, the binding of the ssDNA in the groove differs in detail. For example, LiRecT presents a repetitively kinked five nucleotide/monomer regularity whereas Redβ and HsRAD52 appear to be 4 nucleotides/monomer. Notably in both cases the bound ssDNA is stretched about 1.5 fold, which is the same as ssDNA stretching by RecA/Rad51. The second ssDNA strand is bound to the first by Watson-Crick base pairs with little evidence for extensive binding into a second groove or *trans* interactions with another filament. So it is unlikely that the second strand is stretched before annealing. Consequently, the homology search by SSAPs is likely to be similar to the stretched versus unstretched search mechanism utilized by RecA/Rad51, where an initial match can be found and then expanded as the second strand is zipped into position through Watson–Crick pairing^20^. The evidence for a *cis*-based zipping mechanism concords with observations from atomic force microscopy and optical tweezer single-molecule studies with Redβ, which also revealed a substantial increase in complex stability upon the annealing of ~10 bases (now revealed to be dimerization of Redβ) and a transition to a remarkably stable complex, termed a DNA clamp, resistant to 200 pN of pulling force^11,21^. The basis for a DNA clamp is evident in both of the new bacteriophage filaments, however apparently involving different secondary structural elements that move to secure the DNA after annealing. Once again, the principle appears to be the same however the details are different.

## Outlook

The perception that all SSAPs are ancestrally anchored in the Rad52 superfamily promotes functional implications. Notably, helical filaments have not been reported for RAD52 rather only rings that may be heptamers^8^ or undecamers^9,10^. Despite this evidence that RAD52 multimerization is flexible, rather than a *cis*-zipping mechanism, ring-to-ring *trans*-annealing models have been favoured^19,22^. In light of the new SSAP structures, a reappraisal of the RAD52-annealing mechanism may be rewarding.

Recent progress with the simpler HR Redβ mechanism^23,24^ could also illuminate Rad52 action. Both Rad52 and Redβ annealing domains, which like all members of the Rad52 SSAP superfamily occupy ~180 amino acids at the N-terminus, are insufficient for HR and protein–protein interactions with their C-terminal regions are required^23,25^. One of these interactions involves the major cellular single-strand binding protein, termed replication protein A (RPA) in eukaryotes and single-strand binding (SSB) in prokaryotes^26^. For eukaryotic Rad52, the RPA interaction with a specific Rad52 C-terminal region was defined some time ago^25^. Interaction between the C-terminus of λ phage Redβ and *E.coli* SSB was recently identified by inspired deduction^27^. Concomitantly the first functional evidence for SSB contribution to phage SSAP-mediated HR was reported^28^. This emergent commonality involving eukaryotic Rad52/RPA and prokaryotic phage SSAPs/SSB is another indicator that Rad52 and phage SSAP HR mechanisms are related. Consequentially, now that the Rad52 SSAP superfamily is secured, a new light is cast on Rad52 action and the vast diversity of prokaryotic SSAPs can be confidently evaluated for structural and mechanistic variations around a central theme.

## Methods

For HsRad52 (P43351) we used the PDB structure with ID 1kn0 PMID12191481. For the remaining proteins with IDs Q12223, Q9MC33, P04892, K7P860, Q6XQB4, P03698, UPI00004B3CF7, UPI00006DD4A7, Q92FL9, P33228, Q9T172 we predicted their structure using AlphaFold (version v2.0.1 with the full_dbs option for all but Q92FL9, for which we used version 2.2). Structures were aligned against HsRAD52 79–156. To search for proteins with a similar fold to Rad52, we submitted the HsRAD52 79–156 monomers to Foldseek.

## References

[1] Mortensen UH, Lisby M, Rothstein R (2009). Rad52. Curr. Biol..

[2] Newing T (2022). Redβ177 annealase structure reveals details of oligomerization and λ Red-mediated homologous DNA recombination. Nat. Commun..

[3] Caldwell BJ (2022). Structure of a RecT/Redβ family recombinase in complex with a novel duplex intermediate of DNA annealing. Nat. Commun..

[4] Piazza A, Heyer WD (2019). Homologous recombination and the formation of complex genomic rearrangements. Trends Cell Biol..

[5] Bell JC, Kowalczykowski SC (2016). RecA: regulation and mechanism of a molecular search engine. Trends Biochem. Sci..

[6] Iyer LM, Koonin EV, Aravind L (2002). Classification and evolutionary history of the single-stranded annealing proteins, RecT, Redbeta, Erf and RAD52. BMC Genom..

[7] Passy SI, Yu X, Li Z, Radding CM, Egelman EH (1999). Rings and filaments of beta protein from bacteriophage lambda suggest a superfamily of recombination proteins. Proc. Natl Acad. Sci. USA.

[8] Stasiak AZ (2000). The human Rad52 protein exists as a heptameric ring. Curr. Biol..

[9] Singleton MR, Wentzell LM, Liu Y, West SC, Wigley DB (2002). Structure of the single-strand annealing domain of human RAD52 protein. Proc. Natl Acad. Sci. USA.

[10] Kagawa W (2002). Crystal structure of the homologous-pairing domain from the human Rad52 recombinase in the undecameric form. Mol. Cell.

[11] Erler A (2009). Conformational adaptability of Redbeta during DNA annealing and implications for its structural relationship with Rad52. J. Mol. Biol..

[12] Lopes A, Amarir-Bouhram J, Faure G, Petit MA, Guerois R (2010). Detection of novel recombinases in bacteriophage genomes unveils Rad52, Rad51 and Gp2.5 remote homologs. Nucleic Acids Res..

[13] Matsubara K, Malay AD, Curtis FA, Sharples GJ, Heddle JG (2013). Structural and functional characterization of the Redβ recombinase from bacteriophage λ. PLoS ONE.

[14] Jumper J (2021). Highly accurate protein structure prediction with AlphaFold. Nature.

[15] Guo HB (2022). AlphaFold2 models indicate that protein sequence determines both structure and dynamics. Sci. Rep..

[16] Wu Y, Sugiyama T, Kowalczykowski SC (2006). DNA annealing mediated by Rad52 and Rad59 proteins. J. Biol. Chem..

[17] Ploquin M (2008). Functional and structural basis for a bacteriophage homolog of human RAD52. Curr. Biol..

[18] van Kempen, M. et al. Foldseek: fast and accurate protein structure search. Preprint at *bioRxiv*10.1101/2022.02.07.479398v1 (2022).

[19] Saotome M (2018). Structural basis of homology-directed DNA repair mediated by RAD52. iScience.

[20] Klapstein K, Chou T, Bruinsma R (2004). Physics of RecA-mediated homologous recognition. Biophys. J..

[21] Ander M, Subramaniam S, Fahmy K, Stewart AF, Schäffer E (2015). A single-strand annealing protein clamps DNA to detect and secure homology. PLoS Biol..

[22] Grimme JM (2010). Human Rad52 binds and wraps single-stranded DNA and mediates annealing via two hRad52–ssDNA complexes. Nucleic Acids Res..

[23] Subramaniam S (2016). DNA annealing by Redβ is insufficient for homologous recombination and the additional requirements involve intra- and inter-molecular interactions. Sci Rep..

[24] Caldwell BJ, Bell CE (2019). Structure and mechanism of the Red recombination system of bacteriophage λ. Prog. Biophys. Mol. Biol..

[25] Park MS, Ludwig DL, Stigger E, Lee SH (1996). Physical interaction between human RAD52 and RPA is required for homologous recombination in mammalian cells. J. Biol. Chem..

[26] Lin Y (2008). Engineering of functional replication protein a homologs based on insights into the evolution of oligonucleotide/oligosaccharide-binding folds. J. Bacteriol..

[27] Caldwell BJ (2019). Crystal structure of the Redβ C-terminal domain incomplex with λ Exonuclease reveals an unexpected homology with λ Orf and aninteraction with Escherichia coli single stranded DNA binding protein. Nucleic Acids Res..

[28] Yin J (2019). Single-stranded DNA-binding protein and exogenous RecBCD inhibitors enhance phage-derived homologous recombination in *Pseudomonas*. iScience.

